# Non-coding RNAs as diagnostic biomarkers for preeclampsia: a systematic review and meta-analysis

**DOI:** 10.1186/s12884-025-08116-8

**Published:** 2025-09-27

**Authors:** Jiayu Liu, Qipeng Zhao, Yafei Zhu

**Affiliations:** 1 School of Nursing, Ningxia Medical University, No. 1160, Shengli Street, Xingqing District, Yinchuan City, Ningxia China; 2 School of Pharmacy, Key Laboratory of Hui Ethnic Medicine Modernization, Ministry of Education, Ningxia Medical University, No. 1160, Shengli Street, Xingqing District, Yinchuan City, Ningxia China

**Keywords:** Preeclampsia, Non-coding RNA, MiRNA, CircRNA, LncRNA, Diagnostic biomarkers

## Abstract

**Background:**

Preeclampsia (PE), a grave obstetric complication, mandates the expeditious formulation of efficacious early diagnostic strategies. Accumulating evidence suggests that non - coding RNAs (ncRNAs), which are present in maternal circulation and placental tissues, display abnormal expression patterns in patients with PE, underscoring their potential as diagnostic biomarkers. This systematic review and meta - analysis intends to assess the diagnostic accuracy of ncRNAs for the detection of PE.

**Methods:**

A comprehensive search was carried out across seven databases (China National Knowledge Infrastructure [CNKI], Wanfang Database, VIP Database, PubMed, Web of Science, Embase, and the Cochrane Library) up to December 25, 2024, to identify case - control and cohort studies exploring the diagnostic value of ncRNAs in PE. The quality of the studies was evaluated using the Quality Assessment of Diagnostic Accuracy Studies − 2 (QUADAS − 2) tool and the Newcastle - Ottawa Scale (NOS), and publication bias was assessed using Deeks’ funnel plot. The pooled sensitivity (SEN), specificity (SPE), diagnostic odds ratio (DOR), and area under the curve (AUC) were computed using Review Manager 5.4 and Meta - DiSc 1.4.

**Results:**

Among the 2,201 identified studies, 40 fulfilled the inclusion criteria for qualitative synthesis. Forty - eight ncRNAs showed diagnostic potential, including 25 microRNAs (miRNAs), 9 long non - coding RNAs (lncRNAs), and 6 circular RNAs (circRNAs). The pooled sensitivity and specificity were 80% (95% confidence interval [CI]: 76–84%) and 82% (95% CI: 79–85%), respectively. Single miRNA assays presented superior diagnostic performance (sensitivity [SEN]: 85%, specificity [SPE]: 85%) in comparison to circRNAs (SEN: 80%, SPE: 79%). Notably, combinatorial panels consisting of 2–3 ncRNAs attained optimal diagnostic performance, with a sensitivity of 91% (95% CI: 88–94%), a specificity of 80% (95% CI: 76–84%), and an area under the curve (AUC) of 0.9418 (standard error [SE] = 0.0152).

**Conclusion:**

Circulating ncRNAs exhibit significant potential as diagnostic biomarkers for PE, with multi-analyte panels providing improved diagnostic accuracy compared to single-marker strategies.

**Supplementary Information:**

The online version contains supplementary material available at 10.1186/s12884-025-08116-8.

## Introduction

Preeclampsia (PE), a pregnancy-specific multisystem disorder, is clinically manifested as de novo hypertension (≥ 140/90 mm Hg) and proteinuria (≥ 300 mg/24 h) occurring after 20 weeks of gestation. It significantly contributes to maternal and perinatal morbidity and mortality on a global scale [1]. With a global incidence spanning from 2 to 8%, PE is estimated to account for 46,000–76,000 maternal fatalities annually [2]. In China, epidemiological data from 2022 indicated a PE incidence of 2.74% and a maternal mortality rate of 4.6% [3]. Although the exact pathogenesis remains incompletely elucidated, the widely recognized “two-stage theory” posits that inadequate trophoblast invasion and subsequent placental hypoperfusion (Stage 1) trigger the systemic release of placental mediators, including growth factors, inflammatory cytokines, and antiangiogenic proteins, ultimately resulting in maternal endothelial dysfunction (Stage 2) [4]. Current clinical management, encompassing antihypertensive therapy, low-dose aspirin prophylaxis, and magnesium sulfate for seizure prevention, primarily focuses on symptom control. Nevertheless, in severe cases, definitive treatment still necessitates pregnancy termination [5]. These therapeutic constraints underscore the pressing requirement for reliable early diagnostic biomarkers to enable timely intervention and enhance clinical outcomes.

In accordance with the criteria set by the American College of Obstetricians and Gynecologists (ACOG) [6], the current diagnosis of PE relies on clinical manifestations emerging after 20 weeks of gestation, including hypertension and proteinuria. Nevertheless, the sensitivity and clinical applicability of these parameters are restricted. Remarkably, around 20% of PE cases do not exhibit significant proteinuria [7], and the collection of 24 - hour urine samples presents compliance difficulties in clinical scenarios. Significantly, placental pathological alterations, such as impaired remodeling of the spiral arteries, can occur as early as 8 weeks of gestation [8], far preceding the onset of clinical symptoms. This diagnostic delay highlights the pressing necessity to identify early molecular biomarkers for timely detection and intervention.

In recent years, non - coding RNAs (ncRNAs) have emerged as promising biomarkers for a variety of diseases, such as tuberculosis, diabetes mellitus, leukemia, and PE. This is attributed to their participation in disease pathogenesis and their stable expression in both serum and tissue samples [9–11]. For instance, dysregulated expression of ncRNAs in maternal serum has been demonstrated to differentiate PE patients from healthy controls as early as the 9th week of gestation [12]. This discovery is consistent with the molecular mechanisms involved in PE pathogenesis, including abnormal vascular endothelial growth factor (VEGF) signaling, characterized by elevated soluble fms-like tyrosine kinase − 1 (sFlt − 1), and oxidative stress resulting from the accumulation of reactive oxygen species (ROS) [13]. Mechanistically, ncRNAs are known to epigenetically regulate these pathways [14], offering a compelling biological foundation for their potential as early diagnostic biomarkers.

Non-coding RNAs (ncRNAs), encompassing microRNAs (miRNAs; approximately 22 nucleotides), circular RNAs (circRNAs), and long non-coding RNAs (lncRNAs; exceeding 200 nucleotides), modulate gene expression without encoding proteins. miRNAs primarily perform their functions by silencing target messenger RNAs (mRNAs) at the post-transcriptional level [15]. In contrast, circRNAs frequently act as miRNA sponges or regulators of RNA-binding proteins [16]. lncRNAs participate in chromatin remodeling and transcriptional regulation [17]. In PE, both placental and circulating ncRNAs display disease-specific dysregulation.

For example, upregulated miR-210 expression leads to placental insufficiency by inhibiting trophoblast invasion via the suppression of the HIF-1α/MMP-9 pathway and impeding mitochondrial respiration through targeting ISCU1/2 iron-sulfur cluster biosynthesis. Moreover, miR-155 intensifies inflammatory responses and undermines endothelial integrity by directly downregulating SOCS1 (suppressor of cytokine signaling 1) [18]. circ_0002814 has been demonstrated to accelerate the progression of PE by sequestering miR-210, thus activating the Notch1/CPEB2 axis and disrupting the FUS/VEGF pathway in trophoblasts [19]. Likewise, lncRNA MEG3 impairs trophoblast migration and apoptosis resistance through the dysregulation of Notch1 signaling [20].

This systematic review integrates the existing evidence regarding the diagnostic utility of ncRNAs in PE and assesses their diagnostic accuracy via meta-analysis.

## Methods

The protocol has been prospectively registered in the PROSPERO database (CRD42025648252).

### Search strategy

This systematic review and meta-analysis were carried out in line with the Preferred Reporting Items for Systematic Reviews and Meta-Analyses (PRISMA) guidelines [21]. A thorough Literature search was executed across seven electronic databases, namely CNKI, Wanfang, VIP, PubMed, Web of Science, Embase, and the Cochrane Library, from their establishment until December 24, 2024. Pre-determined search terms associated with “preeclampsia” and “non-coding RNA” were utilized (refer to Additional file 1).

### Inclusion and exclusion

#### Time period

Studies published from database inception to December 24, 2024, were considered.

#### Study types

Eligible studies incorporated observational research designs, specifically case-control, cross-sectional, and cohort studies, which reported the diagnostic accuracy of ncRNAs for PE. Exclusion criteria comprised reviews, conference abstracts, animal or in vitro studies, and articles devoid of extractable diagnostic performance data. Quantified Data Extraction Criteria: studies eligible for inclusion must present at least one of the following: (1) A complete contingency table (comprising true positives - TP, false positives - FP, false negatives - FN, and true negatives - TN); (2) Data enabling the calculation of a contingency table, including sensitivity, specificity, total sample size (N), and prevalence; (3) Positive predictive value (PPV), negative predictive value (NPV), total sample size (N), and prevalence. Model performance metrics should include the area under the curve (AUC) with a 95% confidence interval (CI) or standard error (SE).

#### Biomarker criteria

Studies that explored either individual or combinatorial ncRNA panels (including miRNAs, lncRNAs, circRNAs, with or without supplementary serum markers) were incorporated, on the condition that ncRNAs functioned as the primary diagnostic biomarkers.

#### Population

The target population encompassed pregnant women aged 18 years or older who were diagnosed with PE in accordance with the standardized criteria of the International Society for the Study of Hypertension in Pregnancy (ISSHP), the American College of Obstetricians and Gynecologists (ACOG), or the World Health Organization (WHO). The control groups were composed of healthy pregnant women matched for gestational age without obstetric complications. Studies involving participants from all countries and ethnic backgrounds were considered eligible, with no limitations imposed on parity. Nevertheless, studies that included participants with significant comorbidities (e.g., chronic hypertension, diabetes mellitus, renal disease, or autoimmune disorders) were excluded. For inclusion, critical methodological details were required, specifically comprehensive reporting of participant inclusion and exclusion criteria, sample collection and processing protocols, laboratory detection techniques, and statistical analysis methods, to guarantee publicly verifiable experimental procedures and data reliability.

### Study screening and data extraction

All retrieved records were imported into NoteExpress version 3.9 for duplicate removal. Two reviewers independently carried out title and abstract screening, followed by full-text evaluation in accordance with the predefined eligibility criteria. Any discrepancies were resolved via discussion and consensus-building.

Data were retrieved regarding the following variables: the first author, the year of publication, the sample size, the gestational age at the time of sampling, the sample type (e.g., blood or placental tissue), the values of true positive (TP), false positive (FP), true negative (TN), and false negative (FN), the area under the curve (AUC), the sensitivity, the specificity, and the diagnostic odds ratio (DOR). For studies with missing or incomplete data, the corresponding authors were contacted through email. Studies with unresolved data-related queries were excluded from the analysis.

### Quality assessment

#### QUADAS-2 tool

The methodological quality of the included studies was evaluated by means of the QUADAS − 2 tool, which assesses four domains: patient selection, index test, reference standard, and flow and timing. Two reviewers independently rated the bias risk and applicability concerns in each domain as “low”, “high”, or “unclear”. Any discrepancies between reviewers were resolved via discussion and consensus.

#### The Newcastle–Ottawa Scale (NOS)

The Newcastle–Ottawa Scale (NOS) is a star-based tool specifically developed for evaluating the methodological quality of observational studies. It consists of two parallel sets of criteria: one for case–control studies and the other for cohort studies, with a maximum of nine stars assignable for each set. Studies that accumulate ≥ 7 stars are regarded as being of high quality; those with 5–6 stars are classified as having moderate quality; and those with < 5 stars are considered to be of low quality.

### Data synthesis and statistical analysis

Data analysis was conducted using RevMan 5.4, R version 4.3.1 (meta package), and Meta-DiSc 1.4. Heterogeneity was quantified via the I² statistic. In accordance with Cochrane guidelines [22], random-effects models were employed when the I² value exceeded 50%, whereas fixed-effects models were utilized for I² values of 50% or lower. Pooled sensitivity, specificity, positive likelihood ratio (PLR), negative likelihood ratio (NLR), and diagnostic odds ratio (DOR) were computed. Summary receiver operating characteristic (SROC) curves, along with their corresponding area under the curve (AUC) values, were constructed. Publication bias was evaluated using Deeks’ funnel plot asymmetry test, with a significance threshold established at *p* ≤ 0.1.

## Result

### Characteristics of included studies

**Fig. 1:**
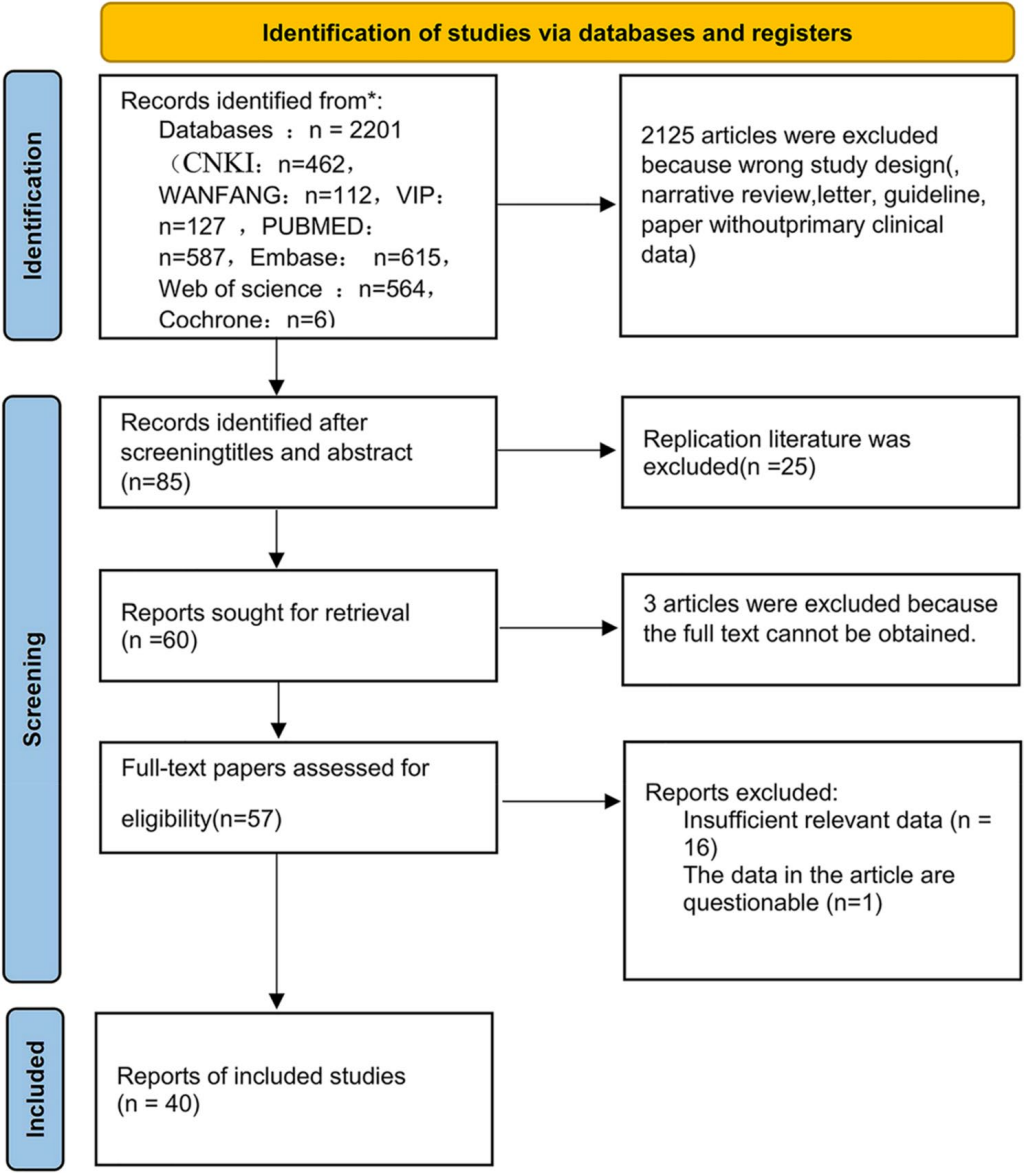
PRISMA flowchart showing study selection process

The process of study selection is presented in the PRISMA flowchart (Fig. 1). Among the 2,201 records initially identified via database searches, 2,116 articles were excluded during the screening of titles and abstracts owing to their irrelevance (e.g., non-diagnostic studies, animal/cell models). A full-text evaluation of 85 potentially eligible articles resulted in the exclusion of 45 studies for the following reasons: incomplete diagnostic data (*n* = 28), inaccessible full texts (*n* = 12), or duplication (*n* = 5). This procedure led to the inclusion of 40 studies for the final analysis (miRNA: *n* = 23; lncRNA: *n* = 11; circRNA: *n* = 6).

The included studies, published from 2015 to 2024, analyzed samples collected between 2012 and 2023. Geographically, 34 studies (85%) were carried out in China, while the remaining 6 studies originated from Egypt (*n* = 2), Australia (*n* = 1), Turkey (*n* = 1), the Czech Republic (*n* = 1), and the United States (*n* = 1).

In the included studies, the sample sizes in the control groups ranged from 19 to 594, and the sample sizes in the PE groups ranged from 21 to 212. All 40 studies utilized quantitative real-time PCR (qRT - PCR) for the quantification of ncRNA. Blood samples were the predominant source of biospecimens (90%, 36/40 studies), and placental tissue was analyzed in 4 studies (10%). The key characteristics of the included studies are summarized in Table 1.

**Table 1:** Baseline characteristics of included studies (*n* = 40)

	First author	Country		Publication Time	Sample Size(control)	Sample Size(PE)	Gestational Week	Literature type	Reference
1	Y-G Zhang	China	qRT-PCR	2016	41	41	8–20	Prospective study	[23]
2	Yonggang Zhang	China	qRT-PCR	2020	64	64	8–20	Prospective study	[24]
3	Min Jiang	China	qRT-PCR	2018	30	30	< 20	Prospective study	[25]
4	Dan Chen	China	qRT-PCR	2021	30	24	11–12	Prospective study	[26]
5	QIng Ye	China	qRT-PCR	2021	42	42	22–28	Retrospective study	[27]
6	Dan Chen	China	qRT-PCR	2022	29	20	11–13	Prospective study	[28]
7	Haixia Gao	China	qRT-PCR	2024	124	124	29–37	Retrospective study	[29]
8	Yingjie Zhang	China	qRT-PCR	2024	100	124	28–36	Retrospective study	[30]
9	Chunxai Huo	China	qRT-PCR	2023	247	288	35–40	Retrospective study	[31]
10	Danling Chen	China	qRT-PCR	2018	35	70	34–41	Retrospective study	[32]
11	Ye Song	China	qRT-PCR	2024	60	112	32–41	Retrospective study	[33]
12	Dongmei Zhang	China	qRT-PCR	2021	48	87	24	Retrospective study	[34]
13	Zhengjiao Liang	China	qRT-PCR	2023	73	72	35–39	Retrospective study	[35]
14	Ahmed Abbas Mona	Egypt	qRT-PCR	2023	75	75	30–39	Retrospective study	[36]
15	Haiwei Yu	China	qRT-PCR	2023	108	108	28–36	Retrospective study	[37]
16	Zhiming Liu	China	qRT-PCR	2024	106	212	NR	Retrospective study	[38]
17	Sanqiang Niu	China	qRT-PCR	2020	60	60	20–41	Retrospective study	[39]
18	Hongxia Wan	China	qRT-PCR	2021	161	37	14–25	Retrospective study	[40]
19	HuiXian Kang	China	qRT-PCR	2023	540	60	10–14	Retrospective study	[41]
20	Aiping Zhang	China	qRT-PCR	2022	135	95	11–13	Retrospective study	[42]
21	Xunmei Sun	China	qRT-PCR	2022	50	102	27–39	Retrospective study	[43]
22	Yong Wang	China	qRT-PCR	2021	594	34	16–20	Retrospective study	[44]
23	Huijuan Han	China	qRT-PCR	2019	89	48	15–19	Retrospective study	[45]
24	Qin Wang	China	qRT-PCR	2015	36	36	20–24	Retrospective study	[46]
25	Yan Jin	China	qRT-PCR	2021	34	68	23–35	Retrospective study	[47]
26	Xiaowei Wu	China	qRT-PCR	2023	79	79	28–34	Retrospective study	[48]
27	Kun Dong	China	qRT-PCR	2020	40	20	20–40	Retrospective study	[49]
28	Wenwen Ning	China	qRT-PCR	2022	32	58	9–13	Prospective study	[50]
29	Xin He and Danni Ding	China	qRT-PCR	2022	50	126	20–40	Prospective study	[51]
30	Ilona Hromadnikova	Czech Republic	qRT-PCR	2017	58	21	10–13	Prospective study	[52]
31	Siqi Bao	China	qRT-PCR	2022	29	36	10–14	Prospective study	[53]
32	Xiaopeng Hu	China	qRT-PCR	2018	110	34	24	Retrospective study	[54]
33	Bai Yuxiang	China	qRT-PCR	2018	42	30	14–19	Retrospective study	[55]
34	Edward E. Winger	USA	qRT-PCR	2015	19	39	11–13	Retrospective study	[56]
35	Yonggang Zhang	China	qRT-PCR	2017	52	26	12–20	Retrospective study	[57]
36	CaroleAnneWhigham	Austraia	qRT-PCR	2020	196	34	35–37	Prospective study	[58]
37	Xiaolan Zhao	China	qRT-PCR	2021	100	112	15–16	Retrospective study	[59]
38	Tarek M K Motawi	Egypt	qRT-PCR	2018	100	100	24	Retrospective study	[60]
39	Meryem Hocaoglu	Turkey	qRT-PCR	2019	28	23	33	Retrospective study	[61]
40	Chunzhi Sheng	China	qRT-PCR	2020	200	200	37	Retrospective study	[62]

### Quality assessment results

#### QUADAS-2

**Fig. 2:**
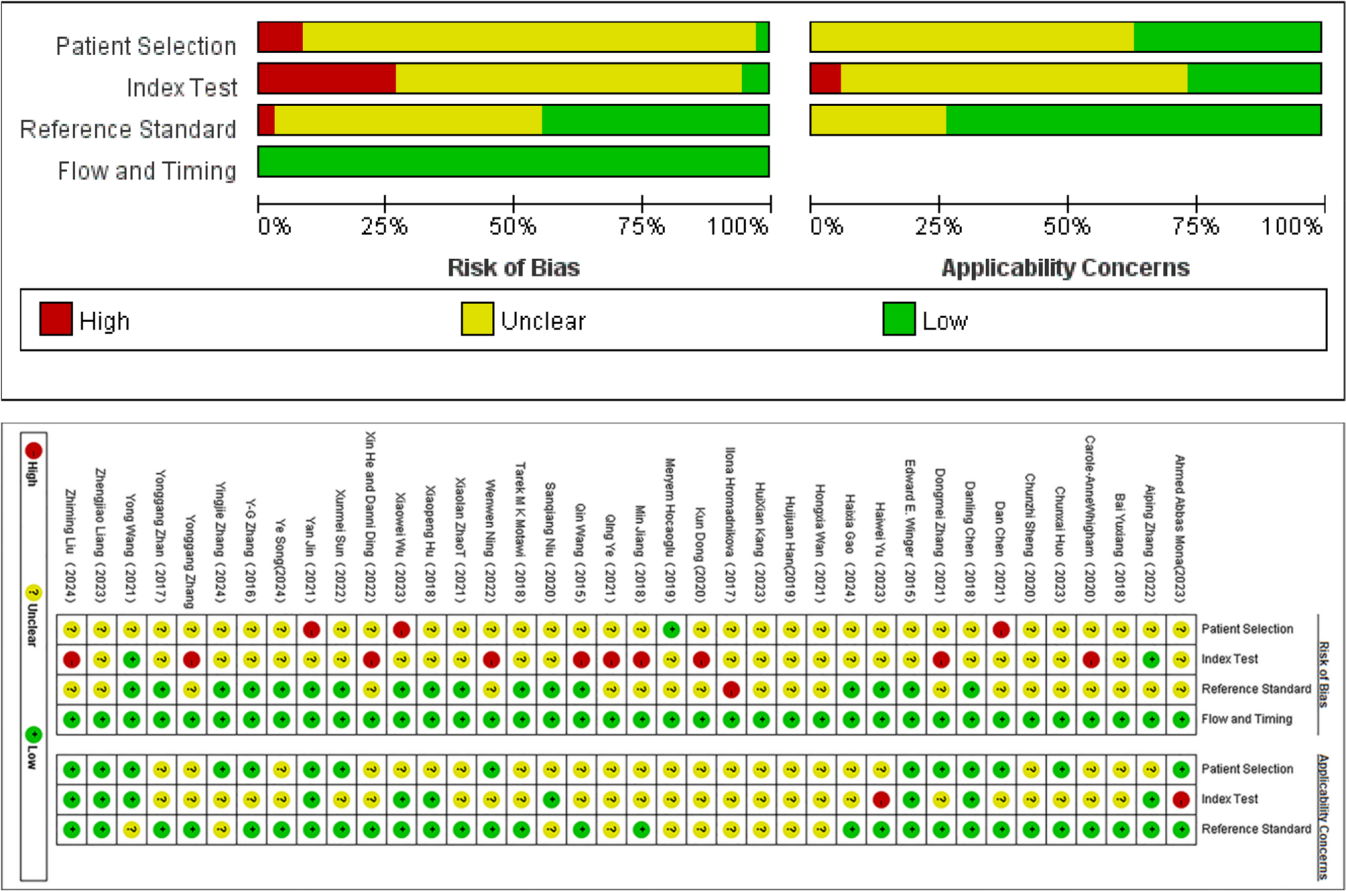
Quality Assessment of Diagnostic Accuracy Studies (QUADAS)−2 assessment for risk of bias and applicability. Red, yellow and green indicate high, unclear and low risk respectively

Quality assessment via the QUADAS-2 tool uncovered substantial methodological issues. Within the patient selection domain, 97.5% (39/40) of the studies were categorized as having a high or indeterminate risk of bias, mainly attributable to non-consecutive enrollment or the utilization of case-control study designs. In the index test domain, 25% of the articles were deemed to be at high risk owing to the lack of blinding procedures and ambiguous threshold values. Across all studies, the flow and timing domain demonstrated low-risk ratings, suggesting the consistent implementation of reference standards. The detailed risk stratification across the four domains (patient selection, index test, reference standard, flow/timing) is visually presented in Fig. 2.

#### The Newcastle–Ottawa scale (NOS)

The methodological quality of the 40 included studies was evaluated using the Newcastle-Ottawa Scale (NOS). Among the 30 case-control studies (with a maximum of 8 stars), 73.3% (*n* = 22) met the high-quality criteria (≥ 7 stars). Specifically, 9 studies achieved a full score, and 13 studies scored 7 stars. The remaining 8 studies (26.7%) scored 6 stars, indicating moderate quality. All 10 prospective cohort studies (with a maximum of 9 stars) were of high quality (≥ 7 stars). This included 1 study with a maximum score, 5 studies scoring 8 stars, and 4 studies scoring 7 stars. Significantly, all studies attained a score of ≥ 6 stars, and there were no critical flaws in key domains (e.g., case representativeness, control selection), which confirms the robust overall quality of these studies to support reliable conclusions.

### Publication bias analysis

**Fig. 3:**
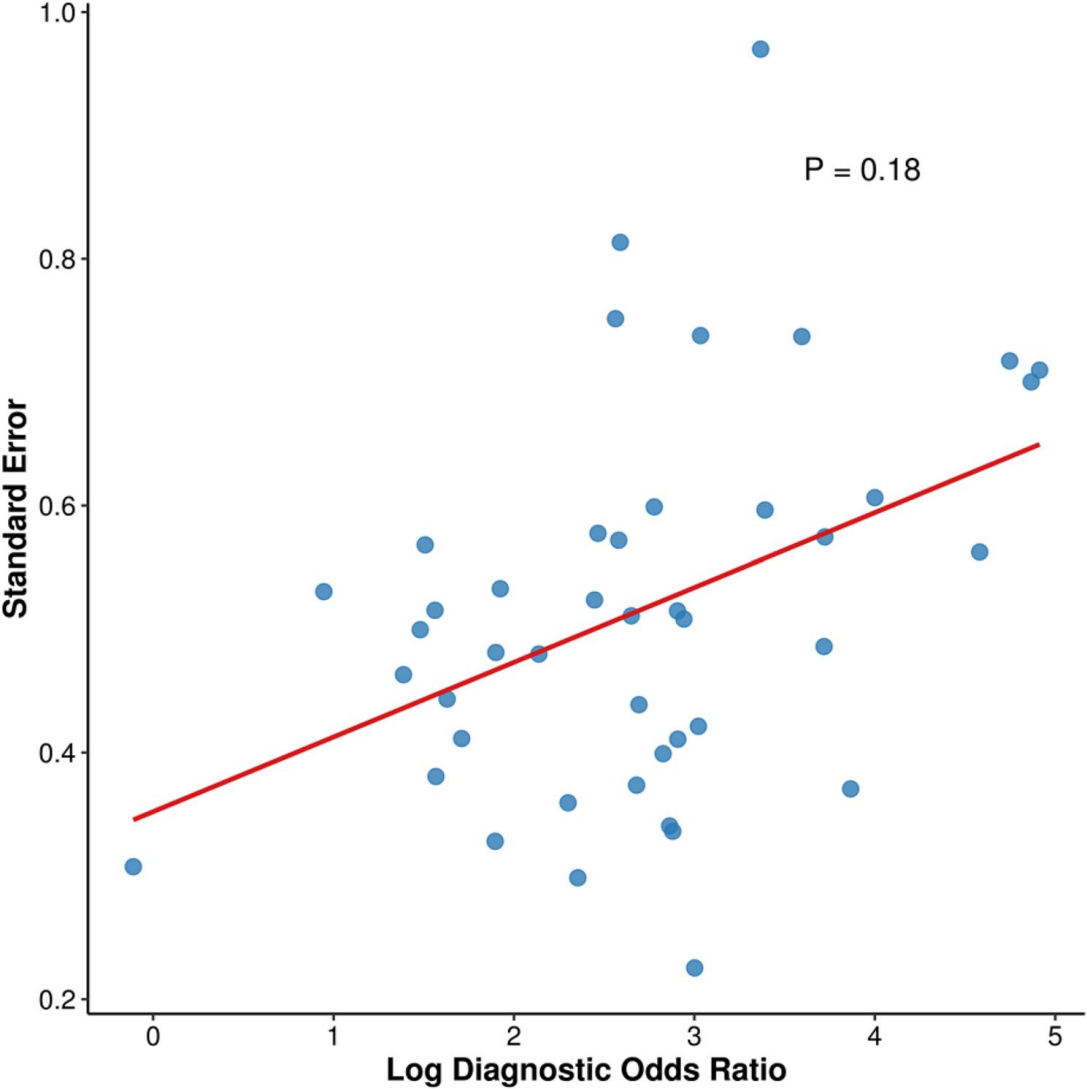
Deeks’ funnel plot assessing publication bias in diagnostic accuracy

Deeks’ funnel plot asymmetry test indicated no substantial publication bias (*p* = 0.18, slope coefficient = 1.32), as evidenced by the symmetrical distribution of effect sizes presented in Fig. 3. This result implies a negligible likelihood of systematic bias among the included studies.

### Diagnostic accuracy of NcRNAs for PE

#### Pooled accuracy of individual NcRNAs

**Fig. 4:**
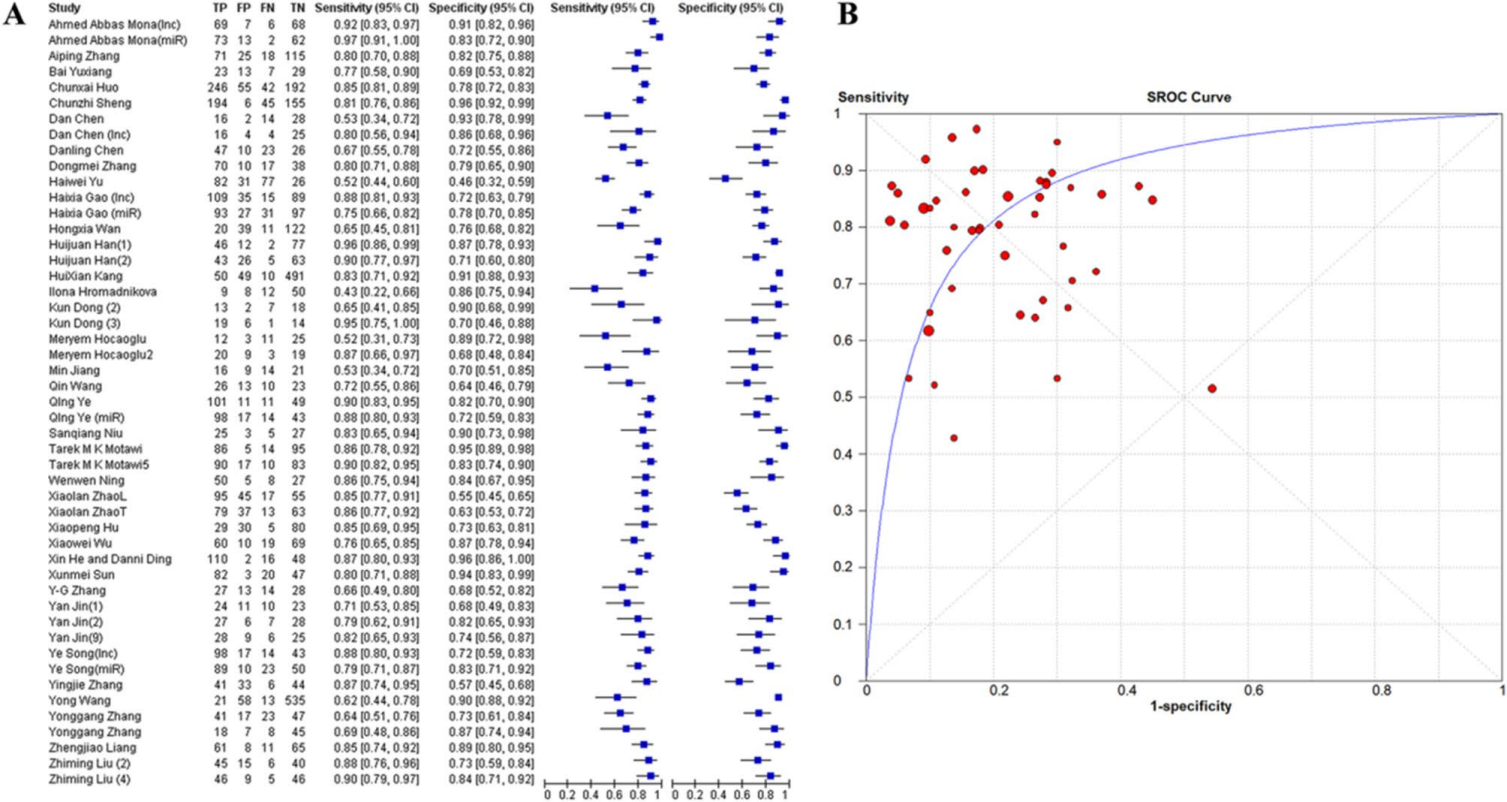
Forest plots(**A**), SROC curves(**B**) for all noncoding RNAs independently. TP (True Positives), FP (False Positives), FN (False Negatives) and TN (True Negatives) were calculated using the reported sensitivity, specificity, total subjects and positive tests

The forty included studies identified forty-eight distinct ncRNAs with diagnostic potential for PE (Table 2). Meta-analysis indicated substantial heterogeneity (I²=81.9%), necessitating the application of a random-effects model. Pooled estimates manifested moderate diagnostic performance, with a sensitivity of 81% (95% confidence interval [CI]: 79–82%), a specificity of 81% (95% CI: 79–82%), and a diagnostic odds ratio (DOR) of 17.196 (95% CI: 12.34–23.97). The area under the summary receiver operating characteristic (SROC) curve was 0.8647 (standard error [SE] = 0.021), with positive and negative Likelihood ratios of 3.94 (95% CI: 3.2–4.8) and 0.26 (95% CI: 0.21–0.32), respectively (Fig. 4; Table 3).

**Table 2:** Detailed characteristics of 48 Non-Coding RNAs

	ncRNA	Author	sample used	Validation method	ncRNA profile	Sensitivity	Specificity	AUC	AUC difference
18	lncRNA-TCL6	Ahmed Abbas Mona	blood	qRT-PCR	> *	0.92	0.91	0.97	0.13
38	miR-200a-3p	Xin He and Danni Ding	blood	qRT-PCR	>	0.87	0.96	0.95	0.11
45	miR-495	Tarek M K Motawi	blood	qRT-PCR	>	0.90	0.83	0.94	0.10
48	miR-206	Chunzhi Sheng	blood	qRT-PCR	>	0.97	0.78	0.94	0.10
17	miRNA-21	Ahmed Abbas Mona	blood	qRT-PCR	< **	0.97	0.83	0.93	0.09
7	lnc-C17orf64-1 ∶ 1	Dan Chen	blood	qRT-PCR	>	0.80	0.86	0.92	0.08
28	miR-200c	Huijuan Han	blood	qRT-PCR	>	0.96	0.87	0.92	0.08
16	lncRNA NORAD	Zhengjiao Liang	blood	qRT-PCR	>	0.85	0.89	0.91	0.07
24	miR-29a-3p	HuiXian Kang	blood	qRT-PCR	>	0.83	0.91	0.90	0.06
26	miR-103a	Xunmei Sun	blood	qRT-PCR	>	0.80	0.94	0.90	0.06
11	lncRNA HIF1A-AS1	Chunxai Huo	blood, plasma	qRT-PCR	<	0.85	0.78	0.88	0.04
14	miR-132-3p	Ye Song	plasma	qRT-PCR	>	0.79	0.83	0.88	0.04
22	miR-195	Sanqiang Niu	blood	qRT-PCR	<	0.83	0.90	0.88	0.04
35	miR-31	Kun Dong	blood	qRT-PCR	<	0.95	0.70	0.88	0.04
13	lncRNA SNHG5	Ye Song	plasma	qRT-PCR	<	0.88	0.72	0.87	0.03
44	miR-494	Tarek M K Motawi	blood	qRT-PCR	>	0.86	0.95	0.87	0.03
25	miR-210	Aiping Zhang	blood	qRT-PCR	>	0.81	0.82	0.86	0.02
21	mir-4443	Zhiming Liu	blood	qRT-PCR	>	0.90	0.84	0.85	0.01
40	circ_0036877	Xiaopeng Hu	blood	qRT-PCR	>	0.85	0.73	0.85	0.01
20	mir-20b	Zhiming Liu	blood	qRT-PCR	>	0.88	0.73	0.84	0
23	miR-101	Hongxia Wan	blood	qRT-PCR	>	0.70	0.76	0.84	0
8	lncRNA DUXAP8	Haixia Gao	blood	qRT-PCR	<	0.88	0.72	0.83	−0.01
15	lncRNAMALAT 1	Dongmei Zhang	blood	qRT-PCR	<	0.81	0.79	0.83	−0.01
9	miR-24-3p	Haixia Gao	blood	qRT-PCR	>	0.75	0.78	0.82	−0.02
2	circCRAMP1L	Yonggang Zhang	blood	qRT-PCR	<	0.64	0.74	0.81	−0.03
4	circ_0025992	Dan Chen	blood	qRT-PCR	>	0.54	0.93	0.81	−0.03
47	miR-116-5p	Meryem Hocaoglu	blood	qRT-PCR	<	0.87	0.68	0.81	−0.03
10	lncRNATCL6	Yingjie Zhang	blood	qRT-PCR	>	0.87	0.57	0.80	−0.04
31	miR-19a	Yan Jin	blood	qRT-PCR	>	0.81	0.74	0.80	−0.04
43	miR-149-5p	Xiaolan Zhao	blood	qRT-PCR	<	0.80	0.85	0.80	−0.04
36	miR-21	Kun Dong	blood	qRT-PCR	<	0.65	0.90	0.79	−0.05
27	miR-18a	Yong Wang	blood	qRT-PCR	<	0.62	0.90	0.78	−0.06
6	miR-206	QIng Ye	blood	qRT-PCR	>	0.71	0.83	0.75	−0.09
12	miRNA-206	Danling Chen	blood	qRT-PCR	>	0.68	0.74	0.75	−0.09
5	lncRNA MALAT1	QIng Ye	blood	qRT-PCR	<	0.74	0.71	0.73	−0.11
42	miR-149-5p	Xiaolan Zhao	blood	qRT-PCR	<	0.73	0.84	0.73	−0.11
19	miR-181a	Haiwei Yu	blood	qRT-PCR	>	0.76	0.71	0.72	−0.12
29	miR-942	Yonggang Zhang	blood	qRT-PCR	<	0.67	0.88	0.72	−0.12
33	miR-210	Yan Jin	blood	qRT-PCR	>	0.80	0.82	0.72	−0.12
37	miR-146b-5p	Wenwen Ning	blood	qRT-PCR	>	0.87	0.84	0.72	−0.12
41	circ_0007121	Bai Yuxiang	plasma	qRT-PCR	<	0.77	0.70	0.72	−0.12
1	circ_101222	Y-G Zhang	blood	qRT-PCR	>	0.66	0.69	0.71	−0.13
34	miR-29b	Xiaowei Wu	blood	qRT-PCR	>	0.76	0.88	0.71	−0.13
30	miR-152	Qin Wang	blood	qRT-PCR	>	0.72	0.64	0.70	−0.14
39	miR-517-5p	Ilona Hromadnikova	blood	qRT-PCR	>	0.43	0.86	0.70	−0.14
46	miR-21-3p	Meryem Hocaoglu	blood	qRT-PCR	<	0.52	0.89	0.69	−0.15
32	miR-126	Yan Jin	blood	qRT-PCR	<	0.71	0.68	0.63	−0.21
3	circRNA − 0004904	Min Jiang	blood	qRT-PCR	>	0.53	0.70	0.62	−0.22

* “>”Indicates that the expression level of this ncRNA is significantly upregulated in women with preeclampsia compared to normal pregnant women

** “<” Indicates that the expression level of this ncRNA is significantly downregulated in women with preeclampsia compared to non-pregnant healthy women

AUC difference: [ncRNA AUC] - [sFlt-1/PlGF reference]

**Table 3:** Diagnostic performance of individual NcRNAs

ncRNA type	Studies(*n*)	Sensitivity (%)	Specificity (%)	DOR	AUC
miRNA	23	80(78–82)	83(81–85)	18.438	0.880
lncRNA	11	85(82–87)	79(76–81)	20.096	0.905
circRNA	6	66(60–872)	74(68–78)	6.163	0.776

#### Diagnostic accuracy of single MiRNAs

**Fig. 5:**
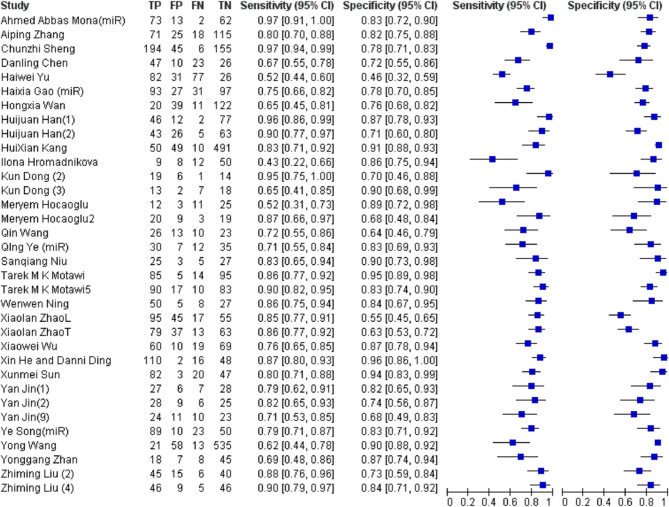
Forest Plot of miRNAs as single Diagnostic Biomarkers for Preeclampsia. TP (True Positives), FP (False Positives), FN (False Negatives) and TN (True Negatives) were calculated using the reported sensitivity, specificity, total subjects and positive tests

Twenty-three studies encompassing 4,898 participants conducted an evaluation of miRNA-based diagnostics. The pooled analysis indicated a moderate level of diagnostic performance, characterized by a sensitivity of 80% (95% CI 78–82%), a specificity of 83% (95% CI 81–85%), an area under the curve (AUC) of 0.88 (SE = 0.018), and a diagnostic odds ratio (DOR) of 18.438 (95% CI 12.7–26.8). The positive likelihood ratio (PLR) and negative likelihood ratio (NLR) were 4.08 (95% CI 3.24–5.14) and 0.25 (95% CI 0.21–0.31), respectively (Fig. 5).

#### Diagnostic accuracy of single LncRNAs

**Fig. 6:**
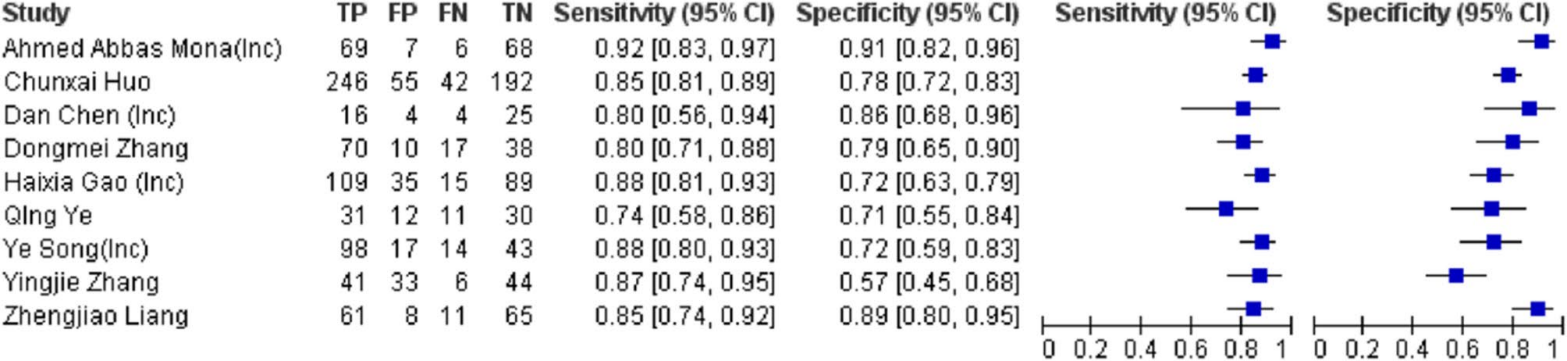
Forest Plot of lncRNAs as single Diagnostic Biomarkers for Preeclampsia. TP (True Positives), FP (False Positives), FN (False Negatives) and TN (True Negatives) were calculated using the reported sensitivity, specificity, total subjects and positive tests

Eleven studies encompassing 1,910 participants conducted an assessment of lncRNA biomarkers, revealing that lncRNA biomarkers exhibited a higher sensitivity (85%, 95% CI 82–87%) in comparison to other ncRNA classes. The specificity was determined to be 79% (95% CI 76–81%), with an area under the curve (AUC) of 0.905 (SE = 0.015) and a diagnostic odds ratio (DOR) of 20.096 (95% CI 14.3–28.2). The positive likelihood ratio (PLR) and negative likelihood ratio (NLR) were 3.82 (95% CI 3.1–4.7) and 0.2 (95% CI 0.16–0.25), respectively (Fig. 6).

#### Diagnostic accuracy of single circRNAs

**Fig. 7:**
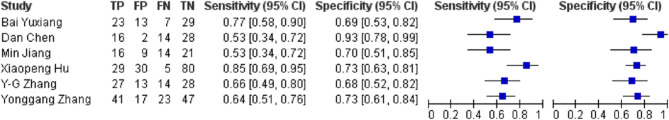
Forest Plot of circRNAs as single Diagnostic Biomarkers for Preeclampsia.TP (True Positives), FP (False Positives), FN (False Negatives) and TN (True Negatives) were calculated using the reported sensitivity, specificity, total subjects and positive tests

Six studies encompassing 540 participants demonstrated Limited diagnostic utility of circRNAs, featuring a sensitivity of 66% (95% confidence interval [CI]: 60–72%), a specificity of 74% (95% CI: 68–78%), an area under the curve (AUC) of 0.7761 (standard error [SE] = 0.032), and a diagnostic odds ratio (DOR) of 6.163 (95% CI: 3.8–10.0). A suboptimal positive likelihood ratio (PLR) of 2.56 (95% CI: 2.0-3.3) and a negative likelihood ratio (NLR) of 0.74 (95% CI: 0.65–0.84) imply restricted clinical applicability (Fig. 7).

#### Combinatorial NcRNA panels

**Fig. 8:**
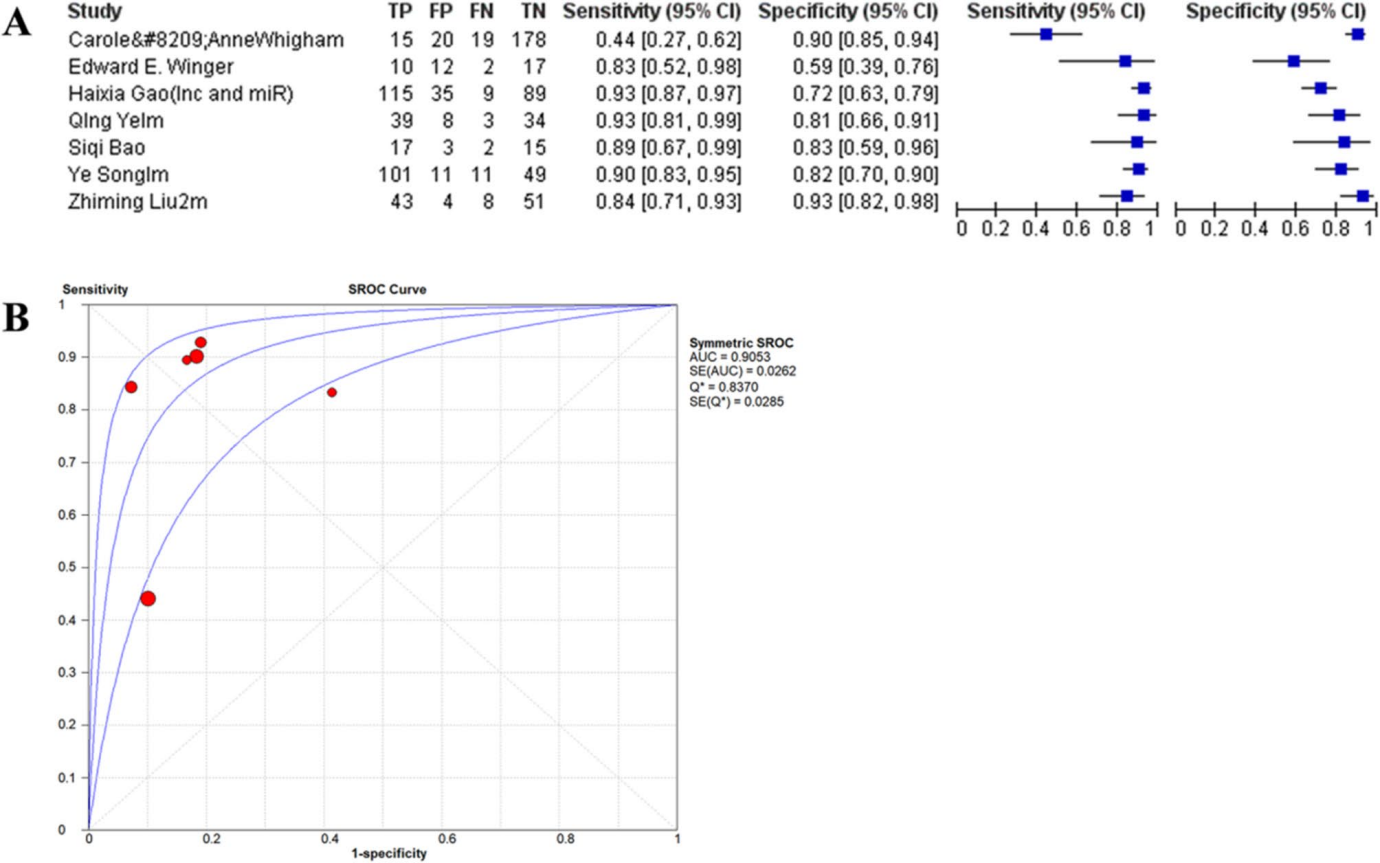
Forest plot(**A**) and SROC curve(**B**) for ncRNA-serum biomarker combinations.TP (True Positives), FP (False Positives), FN (False Negatives) and TN (True Negatives) were calculated using the reported sensitivity, specificity, total subjects and positive tests

Six studies conducted an evaluation of multi-ncRNA panels (comprising 2–3 RNAs), attaining a superior level of diagnostic accuracy. The pooled sensitivity was determined to be 91% (95% confidence interval [CI]: 88–94%), and the specificity was 80% (95% CI: 76–84%). The area under the curve (AUC) was 0.9418 (standard error [SE] = 0.012), and the diagnostic odds ratio (DOR) was 25.56 (95% CI: 18.9–34.6). The AUC values of individual panels ranged from 0.872 to 0.945 (Fig. 8).

#### NcRNA combined with serum biomarkers

**Fig. 9:**
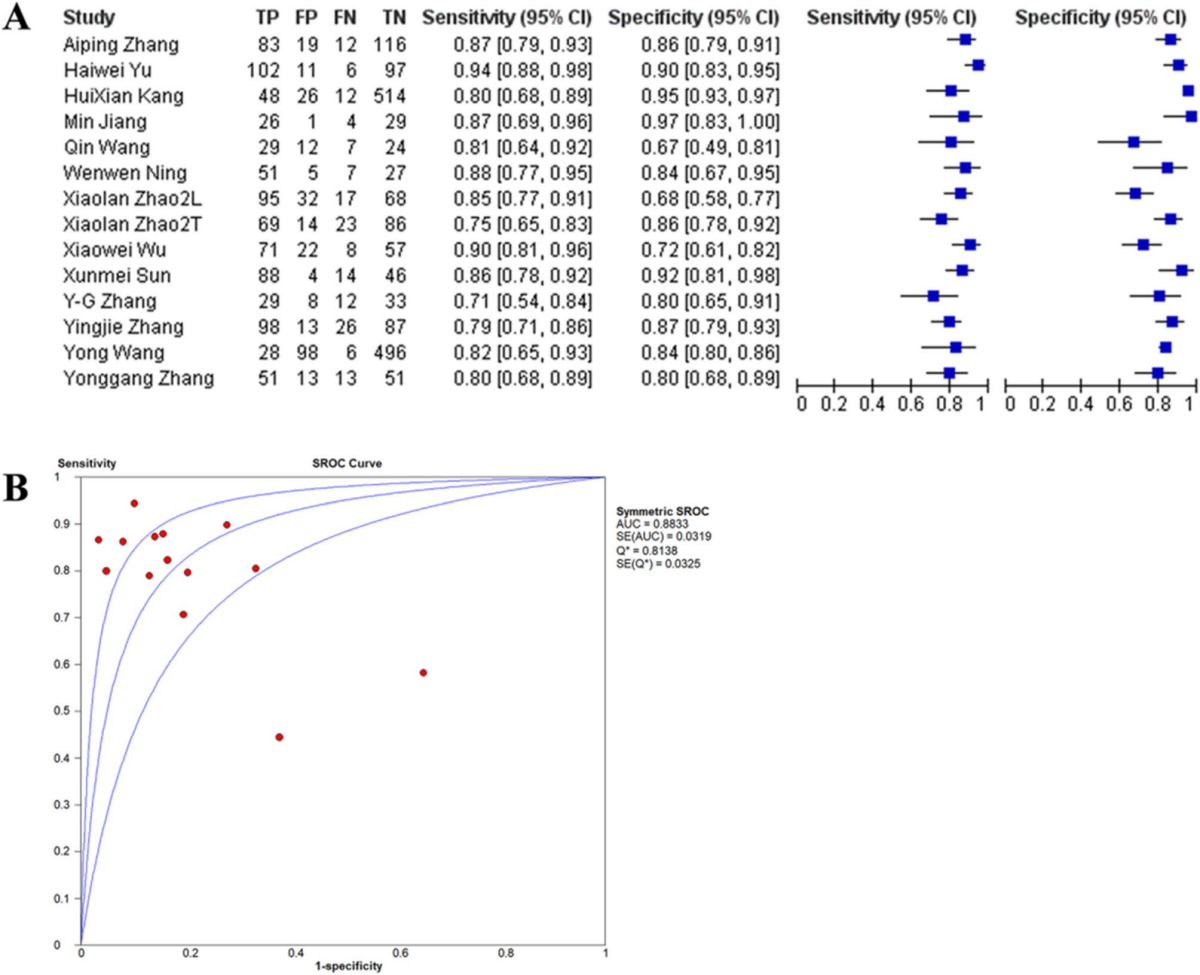
Forest plot(**A**) and SROC curve(**B**) for ncRNA Combined with Serum Biomarkers.TP (True Positives), FP (False Positives), FN (False Negatives) and TN (True Negatives) were calculated using the reported sensitivity, specificity, total subjects and positive tests

Fourteen studies incorporated ncRNAs with established biomarkers (e.g., soluble fms-like tyrosine kinase-1/placental growth factor [sFlt-1/PlGF] ratio, vascular endothelial growth factor-A [VEGF-A]), indicating an improved diagnostic performance. The pooled sensitivity was 85% (95% confidence interval [CI]: 82–88%), the specificity was 87% (95% CI: 84–90%), with an area under the curve (AUC) of 0.9164 (standard error [SE] = 0.017) and a diagnostic odds ratio (DOR) of 20.286 (95% CI: 15.1–27.2). Notably, Wen et al. [47] attained an AUC of 0.929 by utilizing a 5-marker panel (miR-146b-5p + body mass index [BMI] + mean arterial pressure [MAP] + pregnancy-associated plasma protein-A [PAPP-A] + free beta-human chorionic gonadotropin [free β-hCG]) (Fig. 9).

### Differential expression of NcRNAs in PE subtypes

The expression levels of diverse ncRNA types exhibit substantial disparities among distinct subtypes of PE, encompassing severe and mild cases, as well as early-onset and late-onset manifestations.

#### Mild vs. severe PE

Among the 40 included studies, 24 investigated the disparities in non - coding RNA (ncRNA) expression between mild and severe PE. Two studies reported the absence of significant differential expression. Three studies did not present specific ncRNA expression levels; however, they reported significant differences in ncRNA expression between mild and severe PE. Further details are presented in Additional file 2 (sheet1).

#### Early-onset vs. late-onset PE

Two studies failed to present specific data; however, they suggested notable disparities between the two groups. Meanwhile, two other studies reported no significant differences in ncRNA expression between early-onset and late-onset PE. Further details are presented in Additional file 2 (sheet 2).

## Discussion

PE, a life-threatening obstetric complication with an increasing global incidence, emphasizes the pressing requirement for early diagnostic biomarkers to alleviate adverse maternal and fetal outcomes. Although the current diagnostic frameworks that are based on post-20-week hypertension, proteinuria, and end-organ damage are well-established, their Limitations in early detection highlight the necessity for novel biomarkers. ncRNAs, which display disease-specific dysregulation in both placental and maternal circulation as early as 9 weeks of gestation [11], have emerged as promising candidates due to their mechanistic participation in PE pathogenesis.

lncRNAs regulate trophoblast dysfunction and angiogenic imbalance through epigenetic modulation. For instance, lncRNA-MEG3 impairs spiral artery remodeling via the dysregulation of the miR-675/IGF1R axis [63], while lncRNA-ANRIL exacerbates oxidative stress by suppressing Nrf2-mediated antioxidant defenses [64]. circRNAs, despite their structural stability, encounter technical challenges associated with detection standardization [65], which might explain their suboptimal diagnostic performance (AUC = 0.776). In contrast, miRNAs demonstrate superior clinical utility (AUC = 0.873) due to their detectability across multiple tissues (e.g., placenta, serum, and umbilical cord) and their central roles in thromboinflammatory pathways [66].

The substantial risk of bias in patient selection (97.5%) emphasizes the imperative for prospective cohort designs with consecutive enrollment to mitigate spectrum bias. Our meta-analysis indicated significant heterogeneity (I²=81.2%), which was primarily ascribable to ncRNA class and gestational timing. Subgroup analyses yielded the following results:

NcRNA type: Studies on miRNAs manifested higher heterogeneity (I²=83.2%) in comparison to circRNAs (I²=36.9%), reflecting methodological variability in miRNA quantification.

Gestational age: Late-term samples (≥ 35 weeks) displayed lower heterogeneity (I²=46.2%), which is congruent with the convergence of the terminal pathophysiological features of PE.

Sample size: Larger cohorts (*n* > 200) diminished heterogeneity (I²=53.3%), highlighting the significance of adequately powered studies.

Multi-analyte panels, consisting of ncRNAs and serum markers, exhibited superior accuracy (AUC = 0.9418), corroborating the “multi-dimensional biomarker” paradigm [67]. For example, Liu et al. [68] elevated the AUC from 0.85 (single miRNA) to 0.945 by combining miRNAs, while Xu et al. [69] reported exceptional performance (AUC = 0.991) using a circulating miRNA model. These findings underscore the potential of integrating ncRNAs with established biomarkers (e.g., sFlt-1/PlGF) to connect placental and systemic pathways.

Although our analysis classified studies according to PE subtypes (early-/late - onset, mild/severe), none of the included investigations explicitly contrasted the diagnostic performance of ncRNAs across these subtypes. Nevertheless, pooled evidence reveals distinct ncRNA expression patterns between subgroups, suggesting inherent pathophysiological divergences. Existing diagnostic research predominantly concentrates on undifferentiated PE cohorts, despite well-established disparities in clinical trajectories. Early-onset PE (< 34 weeks) is propelled by severe placental malperfusion and defective spiral artery remodeling, whereas late-onset PE (≥ 34 weeks) typically reflects maternal endothelial dysfunction exacerbated by metabolic stressors [70]. Similarly, severe PE is associated with systemic endothelial injury and multi-organ failure, in contrast to the localized placental pathology observed in mild PE [71].

Notably, a seminal review demonstrated subtype-specific variations in oxidative stress pathways-severe PE is characterized by heightened systemic oxidative damage, while early-onset PE exhibits a placental-specific redox imbalance [72]. These mechanistic disparities likely underlie the observed ncRNA expression divergences.

**Fig. 10:**
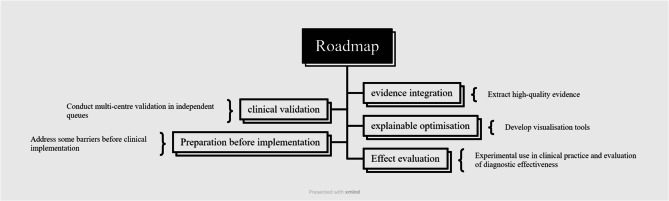
Clinical translation roadmap for ncRNA Biomarkers

Clinical Translation Roadmap:

Fig. 10 Clinical Translation Roadmap for ncRNA Biomarkers.

### Limitations and future directions

Despite promising results, several limitations warrant caution:

#### Geographic bias

The predominance of included studies (85%) originating from China may compromise the generalizability of our findings. Due to potential ethnic, environmental, and healthcare system differences, the diagnostic accuracy reported here may not be directly extrapolable to other populations.

#### Technical variability

The absence of standardized ncRNA detection protocols, such as adherence to MIQE guidelines for qRT-PCR, introduces potential inconsistencies in results.

#### Design limitations

Risks Inherent to Retrospective Design: “Moreover, the retrospective design is inherently accompanied by methodological constraints. Firstly, it presents a risk of selection bias, since the inclusion of participants and the availability of data were contingent upon historical records rather than prospective enrollment based on standardized criteria. Secondly, the unblinded assessment of outcomes (which is prevalent in retrospective chart reviews) gives rise to a potential for observer/reporting bias. Lastly, retrospective studies frequently lack meticulous control for unmeasured confounders or temporal factors. Collectively, these limitations influence the robustness of causal inferences and underscore the necessity for prospective validation.

#### Inability to conduct subgroup analysis 

The retrospective characteristic of this study, in conjunction with the intrinsic heterogeneity in data collection (e.g., variable gestational ages at the time of sampling, diverse geographical contexts, and differing clinical protocols), precluded the conduct of meaningful subgroup analyses. This limitation substantially constrains our capacity to explore crucial biological or clinical variations. Specifically, we were unable to examine whether there were substantial differences in ncRNA dynamics or associations across key maternal demographic factors, distinct study methodologies, or specific gestational intervals. Consequently, potentially significant subgroup - specific effects, such as variations associated with early versus late gestation or regional healthcare practices, may remain undiscovered, thereby restricting the precision and generalizability of our findings.

#### Emerging NcRNAs

 Exploration of piRNA/PIWI pathways.

#### Different classifications of preeclampsia

 The incapacity to compare the performance of non - coding RNA (ncRNA) across different subtypes of PE is attributable to the data aggregation in primary studies. Future investigations on biomarkers should report accuracy metrics stratified by subtypes to facilitate precision diagnostics.

## Conclusion

In summary, this systematic review and meta-analysis synthesizes the existing evidence regarding the diagnostic potential of ncRNAs for PE. Our findings highlight the substantial promise of ncRNAs as diagnostic biomarkers for PE. Specifically, multi-analyte panels that incorporate two or more ncRNAs exhibit notably high accuracy. Future research ought to prioritize the construction of high-sensitivity predictive models integrating ncRNAs, with the objective of improving early detection and facilitating personalized management of PE. Despite the promising results, standardization and validation of these findings in diverse prospective cohorts are necessary prior to clinical implementation.

## Supplementary Information

Supplementary Material 1: Additional file 1:Database search strategy

Supplementary Material 2.

Supplementary Material 3.PRISMA 2020 Checklist

Supplementary Material 4.

Supplementary Material 5.

Supplementary Material 6.

## Data Availability

No datasets were generated or analysed during the current study.

## Abbreviations

AUC: Area under the curve
CircRNA: CircularRNA
DOR: Diagnostic odds ratio
FN: False negative
FP: False positive
lncRNA: Long non-coding RNA
MiRNA: MicroRNA
ncRNA: Non-coding RNA
NLR: Negative likelihood ratio
PE: Preeclampsia
PLR: Positive likelihood ratio
ROS: Reactive oxygen species
SEN: Sensitivity
SPE: Specificity
SROC: Summary receiver operating characteristic
TN: True negative
TP: True positive
VEGF: Vascular endothelial growth factor
VIP: China Science and Technology Journal Database
