# Supplementary material for: Non-coding RNAs as diagnostic biomarkers for preeclampsia: a systematic review and meta-analysis
Source: BMC Pregnancy Childbirth. 2025 Sep 27;25:949. doi: 10.1186/s12884-025-08116-8 (PMC12476626; doi:10.1186/s12884-025-08116-8)
Supplement: Supplementary file 1 — Supplementary Material 1: Additional file 1:Database search strategy [file 12884_2025_8116_MOESM1_ESM.docx]

Additional file 1:Database search strategy

| database | blaintext |
| --- | --- |
| CNKI | SU=('子痫前期' OR '妊娠高血压' OR '先兆子痫')  AND SU=('非编码RNA' OR 'miRNA' OR '微小RNA' OR 'lncRNA' OR '长链非编码RNA' OR 'circRNA' OR '环状RNA')  AND SU=('诊断' OR '标志物' OR '灵敏度' OR '特异度' OR 'ROC曲线') |
| Wanfang | 主题:("子痫前期" OR "妊娠高血压综合征")  主题:("非编码RNA" OR "miRNA" OR "lncRNA" OR "circRNA")  主题:("诊断" OR "生物标志物" OR "灵敏度" OR "ROC曲线") |
| VIP | 题名或关键词=('子痫前期' + '妊娠高血压') ('非编码RNA' + 'miRNA' + 'lncRNA' + 'circRNA') * ('诊断' + '标志物' + '灵敏度') |
| PubMed | 1."Preeclampsia"[Mesh]OR"Pre-Eclampsia"[tiab] OR"PregnancyToxemia"[tiab]OR "Hypertension, Pregnancy-Induced"[tiab]  2. "RNA, Untranslated"[Mesh] OR "MicroRNAs"[Mesh] OR "RNA, Long Noncoding"[Mesh] OR "RNA, Circular"[Mesh] OR ncRNA[tiab] OR miRNA[tiab] OR lncRNA[tiab] OR circRNA[tiab]  3."Diagnosis"[Mesh] OR "Diagnostic Tests,Routine"[Mesh]OR"Biomarkers"[Mesh] OR "Sensitivity and Specificity"[Mesh] OR diagnos*[tiab] OR biomarker*[tiab] OR ROC[tiab] OR "AUC"[tiab]   1. 1 AND 2 AND 3 |
| Web of Science | TS=((preeclampsia OR "pre-eclampsia" OR "pregnancy toxemia")  AND ("noncoding RNA" OR ncRNA OR miRNA OR lncRNA OR circRNA OR "exosomal RNA")  AND (diagnos* OR biomarker* OR sensitivity OR specificity OR "ROC curve" OR AUC)) |
| Embase | 1. 'preeclampsia'/exp OR 'pre-eclampsia':ti,ab,kw OR 'pregnancy toxemia':ti,ab,kw  2. 'noncoding RNA'/exp OR 'microRNA'/exp OR 'long noncoding RNA'/exp OR 'circular RNA'/exp OR ncRNA:ti,ab,kw OR miRNA:ti,ab,kw OR lncRNA:ti,ab,kw OR circRNA:ti,ab,kw  3. 'diagnosis'/exp OR 'diagnostic test'/exp OR 'biological marker'/exp OR 'receiver operating characteristic'/exp OR diagnos*:ti,ab,kw OR biomarker*:ti,ab,kw OR 'ROC curve':ti,ab,kw OR AUC:ti,ab,kw   1. 1 AND 2 AND 3 |
| Cochrane | 1. [mh "Preeclampsia"] OR "pre-eclampsia":ti,ab,kw  2. [mh "RNA, Untranslated"] OR [mh "MicroRNAs"] OR [mh "RNA, Long Noncoding"] OR ncRNA:ti,ab,kw OR miRNA:ti,ab,kw  3. [mh "Diagnosis"] OR [mh "Sensitivity and Specificity"] OR diagnos*:ti,ab,kw OR biomarker*:ti,ab,kw  4. 1 AND 2 AND 3 |
